# Adoption of the Revised DHHS Guidelines on Breastmilk Feeding and HIV in the United States: Clinical Practices and Barriers

**DOI:** 10.1093/ofid/ofaf607

**Published:** 2025-09-27

**Authors:** Kelechi Ikeri, Swosti Joshi, Vilmaris Quinones Cardona, Linda Hassouneh, Ogechukwu Menkiti

**Affiliations:** Division of Neonatology, Department of Pediatrics, University of South Alabama Children's and Women's Hospital, Mobile, Alabama, USA; Division of Neonatology, Department of Pediatrics, St Christopher's Hospital for Children, Philadelphia, Pennsylvania, USA; Division of Neonatology, Department of Pediatrics, St Christopher's Hospital for Children, Philadelphia, Pennsylvania, USA; Division of Pediatric Infectious Diseases, Department of Pediatrics, University of South Alabama Children's and Women's Hospital, Mobile, Alabama, USA; Division of Neonatology, Department of Pediatrics, St Christopher's Hospital for Children, Philadelphia, Pennsylvania, USA

**Keywords:** breastfeeding, guidelines, HIV, infant, infectious diseases

## Abstract

**Background:**

Understanding current clinical practices and barriers to the implementation of the updated infant feeding guidelines in perinatal HIV exposure can inform the development of interventions to improve practice in the United States (US).

**Methods:**

Between August and December 2024, we electronically administered a survey to actively practicing neonatologists and pediatric infectious diseases (PID) physicians in the US. We conducted a multivariate logistic regression to assess the association between physician characteristics and breastfeeding support.

**Results:**

Of 389 respondents, 21% were PID specialists, and 79% were neonatologists. More PID specialists (64%) than neonatologists (42%) (*P* < .01) indicated breastmilk from the virally suppressed parent with HIV as a feeding option. A few PID physicians (35%) and neonatologists (28%) practiced at centers with guidelines for feeding breastmilk from a parent with HIV. Concern for HIV transmission was the strongest barrier to supporting breastmilk feeding across both subspecialties and all geographical regions, as reported by 61% of PID specialists and 63% of neonatologists. Neonatologists (adjusted Odds ratio (aOR), 0.47; 95% Confidence Interval (CI), .28–.78), attending physicians with 0–5 years (aOR, 0.49; 95% CI, .27–.87) and 6–10 years of experience (aOR, 0.40; 95% CI, .22–.74) compared to those >20years of experience, and those at nonacademic centers (aOR, 0.35; 95% CI, .21–.58), were less likely to offer breastmilk.

**Conclusions:**

In the United States, concerns for perinatal HIV transmission remain a significant barrier to breastfeeding support among both PID subspecialists and neonatologists. Accordingly, interventions to promote breastfeeding support should target persisting concerns for lactational HIV transmission.

## Teaser

We evaluated the extent of national adoption of the updated infant feeding guideline and identified barriers to implementation among subspecialists in the US. We found that recommendations to support breastfeeding among parents with HIV remain largely unadopted in the US.

Breastfeeding confers substantial health benefits for the maternal-infant dyad. Breast milk contains bioactive molecules that help protect newborns against infection, as well as metabolic and immune-related diseases. Breastfeeding promotes maternal-infant bonding, helps prevent postpartum bleeding and depression, and provides protection against chronic diseases for both mother and baby [1–4].

Breastfeeding guidelines for individuals living with human immunodeficiency virus (HIV) have historically varied between high-income and low- to middle-income countries. For over three decades, the potential risk for mother-to-child transmission (MTCT) guided recommendations against breastfeeding by individuals living with HIV in high-resource countries such as the United States (US) [5, 6]. In a multisite study from 2014 to 2022, few individuals living with HIV in the US and Canada chose to breastfeed [7]. This study also reported a wide variation in institutional policies and clinical management protocols in such cases [7]. Conversely, in low-resource countries, exclusive breastfeeding is recommended for mothers who are adherent to antiretroviral therapy (ART) as limited access to safe drinking water and unaffordability of infant formula contribute to higher infant mortality and morbidity in these settings [8–10].

Several studies, primarily from sub-Saharan Africa, have provided evidence for the low risk of MTCT of HIV among women who breastfeed while receiving effective ART and maintaining sustained viral suppression [11–15]. These findings, together with health equity and cultural considerations, prompted a significant policy shift by the US Department of Health and Human Services (DHHS) in 2023, with revised guidelines on infant feeding for individuals living with HIV [16]. The updated recommendations emphasize patient-centered counseling and shared decision-making between healthcare providers and parents when determining infant feeding practices [16]. Several centers have proposed models and practical approaches to support the implementation of these new guidelines [17, 18]. A recent limited review showed practice variability in uptake among pediatric infectious diseases (PID) physicians [19]. However, a comprehensive evaluation of the integration of key components of the new feeding guideline into clinical practice is lacking. To address this knowledge gap, we assessed the proportion of subspecialists who do not recommend breastmilk as a feeding option. We also evaluated the extent of national adoption of the updated infant feeding guideline and identified barriers to implementation among subspecialists directly involved in infant feeding decision-making in the US.

We use the term “breastfeeding” in this text for consistency with clinical guidelines while acknowledging that “chest feeding”, a term that may be more appropriate for some individuals, is also encompassed in our usage of the term.

## METHODS

### Study Design and Population

We conducted a web-based national survey of actively practicing US-attending neonatologists and PID physicians between August 7 and 31 December 2024. This cross-sectional study followed the Strengthening the Reporting of Observational Studies in Epidemiology (STROBE) guidelines [20] and was approved by the University of South Alabama Institutional Review Board.

### Survey Instrument

Using clinical knowledge and literature review, our team, comprising neonatologists and PID attending physicians, designed a 12-question online survey. This survey assessed three specific domains: (1) current clinical practices related to infant feeding, (2) discharge planning and outpatient follow-up, and (3) barriers to providing breastfeeding support for individuals living with HIV. Participants were asked to rank their top three perceived barriers in order of importance. We also included questions on physician characteristics, including US state of practice, years of practice, and practice setting (academic vs nonacademic) (Supplementary Fig. 1). The survey was piloted in May 2024 with six neonatologists and two PID physicians and subsequently revised based on the feedback received.

### Survey Administration

The survey was distributed to 806 PID attending physicians via the Pediatric Infectious Diseases Society (PIDS) online forum on 7 August 2024, and to 3000 attending neonatologist members of the American Academy of Pediatrics Section on Neonatal-Perinatal Medicine (AAP-SONPM) via email on 18 September 2024, with a follow-up reminder sent on 18 October 2024. Respondents were required to answer all survey questions, and data collection concluded on 31 December 2024.

### Categorization of Variables

The subspecialists were classified into two groups: neonatologists and specialists in PID. The US states were categorized according to the four regions defined by the US Census Bureau: Northeast, Midwest, South, and West [21].

### Statistical Analysis

Descriptive statistics were used for physician characteristics and survey responses. Statistical differences in demographic variables between physician groups were analyzed using Pearson's χ^2^ test.

To assess the association between physician characteristics and breastfeeding support, a multivariable logistic regression analysis was conducted. The outcome variable was the offering of breastmilk from an individual with HIV and undetectable viral load at delivery. Covariates included and adjusted for were physician subspecialty, years of practice, and practice setting (academic vs nonacademic). Measures of association were reported as odds ratio (OR) with corresponding 95% confidence intervals (CI). A *P*-value of < .05 was considered statistically significant. Statistical analyses were conducted using SPSS statistical software, Version 29 (SPSS, Chicago, IL, USA).

## RESULTS

### Participant Characteristics

A total of 397 subspecialty physicians completed the survey, and 389 were currently practicing in the US. Of these, 21% (*n* = 83) were PID specialists, and 79% (*n* = 306) were neonatologists. The survey response rate was 10.5% for PID specialists and 10.3% for neonatologists (which is within the standard response rate of 4–18% for PIDS, and 5.4–15% for AAP-SONPM). Only respondents practicing in the US were included in the final analysis. The characteristics of participants are described in Table 1. PID participants were from 32 states, while neonatologists were from 44 states and the District of Columbia. There was representation from PID and neonatology physicians from all four geographical regions. The years of practice as an attending physician did not differ between the two specialties. Most PID (88%) and neonatology (69%) specialists practiced in academic settings (Table 1).

**Table 1.** Characteristics of Respondents

Variables	Respondents *N* = 397	PID *N* = 87	Neonatology *N* = 310	*P*-value (PID vs neonatology)
Experience as attending physician *n* (%)				
0–5 y	108 (27)	26 (30)	82 (26)	.87
6–10 y	86 (22)	18 (21)	68 (22)	.78
11–15 y	70 (18)	12 (14)	58 (19)	.34
16–20 y	31 (8)	3 (3)	28 (9)	.10
>20 y	102 (25)	28 (32)	74 (24)	.05
Region *n* (%)				
Northeast				
Mid-Atlantic	89 (22)	16 (18)	73 (24)	.80
New England	28 (7)	8 (9)	20 (6)	
Midwest				
East North Central	45 (11)	14 (16)	31 (10)	.19
West North Central	25 (7)	5 (6)	20 (6)	
South				
South Atlantic	63 (16)	10 (12)	53 (17)	.86
East South Central	36 (9)	12 (14)	24 (7)	
West South Central	43 (11)	9 (10)	34 (11)	…
West				
Mountain	16 (4)	2 (2)	14 (5)	.19
Pacific	44 (11)	7 (8)	37 (12)	
International	6 (1)	4 (5)	2 (1)	…
Nonspecified	2 (1)	0 (0)	2 (1)	…
Practice setting *n* (%)				
Academic	290 (73)	76 (88)	214 (69)	<.01
Nonacademic	105 (26)	10 (11)	95 (30)	<.01
Nonspecified	2 (1)	1 (1)	1 (1)	…

Abbreviation: PID, pediatric infectious diseases.

### PID Consultation

Over two-thirds (70%) of PID physicians reported providing inpatient consultation to infants of parents with HIV after birth. The majority of neonatologists across all geographic regions (86%) worked in centers where PID physicians were consulted in such cases (Table 2).

**Table 2.** Physician Feeding Practice and Hospital Guidelines by Geographic Region

Variables	Total	Northeast	Midwest	South	West
**Pediatrics infectious diseases specialists**	*N* = 83	*N* = 24	*N* = 19	*N* = 31	*N* = 9
Offer breastmilk from parent with HIV	53 (64)	15 (63)	13 (68)	17 (55)	8 (89)
Provides inpatient consultation to infant born to parent with HIV	70 (84)	23 (96)	19 (100)	23 (74)	5 (56)
Breastmilk feeding guideline for infant born to parent with HIV in my center	29 (35)	9 (38)	10 (53)	6 (19)	4 (44)
Breastmilk discharge guideline for infant born to parent with HIV in my center	37 (45)	11 (46)	11 (58)	10 (32)	5 (56)
**Has feeding or discharge guideline**	*N* = 37	*N* = 11	*N* = 11	*N* = 10	*N* = 5
Components of discharge guideline:					
Timing of outpatient virologic testing of the infant	37 (100)	11 (100)	11 (100)	10 (100)	5 (100)
Frequency of outpatient virologic testing of the infant	35 (95)	10 (91)	11 (100)	9 (90)	5 (100)
Duration of breastfeeding	26 (70)	8 (73)	7 (64)	8 (80)	3 (60)
Weaning off breastmilk	28 (76)	8 (73)	8 (73)	9 (90)	3 (60)
Avoidance of mixed breastmilk and formula feeding	31 (84)	10 (91)	9 (82)	9 (90)	3 (60)
Guidance on situations warranting discontinuing breastfeeding	36 (97)	11 (100)	10 (91)	10 (100)	5 (100)
Neonatologists	*N* = 306	*N* = 93	*N* = 51	*N* = 111	*N* = 51
Offer breastmilk from parent with HIV	129 (42)*	33 (33)*	27 (55)	42 (38)	27 (48)*
Inpatient consultation by PID in my center	264 (86)	81 (84)	43 (84)	98 (86)	42 (86)
Breastmilk feeding guideline for infant born to parent with HIV in my center	86 (28)	22 (20)	19 (40)	31 (28)	14 (26)
Breastmilk discharge guideline for infant born to parent with HIV in my center	92 (31)	24 (25)	22 (45)	28 (24)	18 (33)
**Has feeding or discharge guideline**	*N* = 104	*N* = 26	*N* = 23	*N* = 34	*N* = 21
Components of discharge guideline:					
PID/HIV physician referral	99 (95)	26 (100)	22 (96)	32 (94)	19 (90)
Timing of outpatient virologic testing of the infant	76 (73)	17 (65)	20 (87)	25 (74)	14 (67)
Duration of breast feeding	27 (26)	6 (23)	5 (22)	11 (32)	5 (24)
Weaning off breastmilk	12 (12)	2 (7)	6 (26)	3 (9)	1 (5)
Nursing technique and breast care	49 (47)	15 (58)	9 (39)	18 (53)	7 (33)
Provision of donor breast milk	9 (9)	1 (4)	4 (17)	3 (9)	3 (14)
Guidance on situations warranting discontinuing breastfeeding	69 (66)	16 (62)	17 (74)	25 (74)	11 (52)

Categorical variables are represented as *n* (%).

^*^ *P* < .05, pediatrics infectious diseases versus neonatology between similar groups.

Abbreviations: PIDS, pediatric infectious diseases; HIV, human immunodeficiency virus.

### Feeding Practice, Guidelines, and Policies

When asked about feeding options for infants born to a parent with HIV and an undetectable viral load at delivery, a significantly greater proportion of PID specialists (64%) than neonatologists (42%) (*P* < .01) indicated breastmilk from the parent with HIV as a feeding option. This divergence in feeding recommendations between PID and neonatology was particularly evident in the Northeast (63% vs 33% respectively; *P* = .02) and the West (89% vs 48% respectively; *P* = .04) (Table 2).

Only a minority of PID physicians (35%) and neonatologists (28%) reported practicing at centers with established guidelines or policies for feeding breastmilk from a parent with HIV. Proportions were highest in the Midwest among both PID (53%) and neonatology (40%) (Table 2).

### Hospital Discharge Process and Outpatient Follow-up

Fewer than half of the PID physicians (45%) and neonatologists (31%) practiced at centers with a standardized discharge process for infants receiving breastmilk from a parent with HIV (Table 2).

Most of the PID physicians working in such centers reported having unit discharge policies that included the majority of the key components: timing (100%) and frequency of outpatient virologic testing of the infant (95%); recommended duration of breastfeeding (70%); guidance on weaning from breastmilk (76%); recommendations to avoid mixed feeding with both breastmilk and formula (84%); and education on situations warranting discontinuation of breastfeeding (97%) (Table 2).

Among neonatologists practicing in such centers, the discharge process most frequently included a referral to a PID/HIV physician (95%), the timing of outpatient virologic testing of the infant (73%), and education on situations warranting discontinuation of breastfeeding (66%). In contrast, fewer centers included the recommended duration of breastfeeding (26%), guidance on weaning from breastmilk (12%), instruction on nursing technique and breast care (47%), or provision of emergency donor breast milk supplies to prevent mixed feeding with breastmilk and formula (9%) (Table 2).

### Barriers to Supporting Feeding of Breastmilk From a Parent With HIV

Concern for perinatal HIV transmission was the leading barrier to supporting breastmilk feeding across both subspecialties and all geographical regions, as reported by 61% of PID specialists and 63% of neonatologists (Table 3). Additional commonly reported barriers among both specialties (PID vs neonatology) were the lack of data on the frequency of virologic testing in breastfeeding infants (36% vs 25%), and the lack of unit policy for feeding breastmilk (24% vs 35%) (Table 3).

**Table 3.** Barriers to Provision of Support for Feeding Breastmilk From Parent With HIV

Variables	Total	Northeast	Midwest	South	West
Pediatrics infectious diseases specialists	*N* = 83	*N* = 24	*N* = 19	*N* = 31	*N* = 9
Concern for risk of transmission	51 (61)	20 (83)	9 (47)	18 (58)	4 (44)
Lack of unit policy for feeding breastmilk	20 (24)	8 (33)	3 (16)	7 (23)	2 (22)
Lack of support from other ID specialists	10 (12)	4 (17)	0 (0)	6 (19)	0 (0)
Lack of support from neonatology	9 (11)	3 (13)	0 (0)	5 (16)	1 (11)
Lack of support from lactation specialists	4 (5)	1 (4)	0 (0)	3 (10)	0 (0)
Limited access to ID specialist follow-up	4 (5)	1 (4)	1 (5)	2 (6)	0 (0)
Lack of data on frequency of virological testing in breastfeeding infant	30 (36)	6 (25)	5 (26)	16 (52)	3 (33)
Lack of access to donor breastmilk	18 (22)	5 (21)	5 (26)	6 (19)	2 (22)
Neonatologists	*N* = 306	*N* = 93	*N* = 51	*N* = 111	*N* = 51
Concern for risk of transmission	203 (63)	66 (61)	31 (60)	79 (72)	27 (54)
Lack of unit policy for feeding breastmilk	113 (35)	35 (29)	19 (36)	34 (28)	25 (53)
Lack of support from PID specialists	44 (19)	17 (17)	8 (15)	14 (13)	5 (11)
Lack of support from other neonatologists	72 (23)	23 (21)	9 (17)	28 (27)	12 (25)
Lack of support from lactation specialists	43 (12)	18 (14)	5 (10)	16 (15)	4 (8)
Limited access to ID follow-up	18 (7)	2 (3)	5 (11)	9 (9)	2 (3)
Lack of data on frequency of virological testing in breastfeeding infant	74 (25)	25 (28)	11 (21)	27 (26)	11 (23)
Lack of access to donor breastmilk	46 (13)	19 (15)	10 (22)	10 (8)	7 (10)

Categorical variables are represented as *n* (%).

Percentages for neonatologists reflect weighted percentages.

Abbreviations: ID, infectious diseases; PID, pediatric infectious diseases; Peds, pediatrics; Neo, neonatology.

### Association of Physician Characteristics With Willingness to Offer Breastmilk From a Parent With HIV

Physician subspecialty, duration of practice, and practice setting were associated with the willingness to offer breastmilk from a parent with HIV. Neonatologists (adjusted Odds ratio [aOR], 0.47; 95% CI, .28–.78), attending physicians with fewer years of experience, specifically those with 0–5 years (aOR, 0.49; 95% CI, .27–.87) and 6–10 years (aOR, 0.40; 95% CI, .22–.74) as well as those practicing in nonacademic settings (aOR, 0.35; 95% CI, .21–.58), were significantly less likely to offer breastmilk from a parent with HIV as a feeding option (Table 4).

**Table 4.** Association of Respondent Characteristics With Willingness to Offer Breastmilk From Parent With HIV

Characteristics	OR (95% CI)	*P*-Value	Adjusted Odds Ratio (95% CI)	*P*-Value
Subspecialty	…	<.01	…	<.01
PID	Reference		Reference	…
Neonatology	0.40 (.24-.65)		0.47 (.28-.78)	…
Duration of practice	…	<.01	…	<.01
0–5 yrs	0.76 (.49–1.19)		0.49 (.27-.87)	…
6–10 yrs	0.58 (.35-.94)		0.40 (.22-.74)	…
11–15 yrs	1.10 (.66–1.85)		0.72 (.38–1.35)	…
16–20 yrs	1.24 (.60–2.59)		1.00 (.43–2.35)	…
>20 yrs	Reference		Reference	…
Practice setting	…	<.01	…	<.01
Academic	Reference		Reference	…
Nonacademic	0.33 (.20-.53)		0.35 (.21-.58)	…

Abbreviations: PID, pediatric infectious diseases; CI, confidence interval.

## DISCUSSION

To our knowledge, this is the first comprehensive study to evaluate adoption, clinical integration, and barriers to implementation of the revised feeding guidelines among both PID specialists and neonatologists practicing in the US. This study was conducted between seven and eleven months following the issuance of the most recently revised feeding guidelines by the DHHS. In this survey, breastfeeding support to parents living with HIV has not been widely implemented or integrated into practice by US subspecialists. Notably, neonatologists, subspecialty physicians with <10 years of clinical experience, and those working in nonacademic settings were less likely to endorse this practice. Lactational HIV transmission risk remains a significant concern and barrier, compounded by the widespread lack of standardized clinical protocols and discharge processes to guide management.

The only feeding option that eliminates the risk of HIV transmission through breastmilk is replacement feeding with properly prepared formula or pasteurized donor human milk [16]. Breastfeeding avoidance was therefore traditionally recommended among individuals living with HIV in high-income countries, as formula feeding is widely available, safe, and sustainable in these settings [5, 6, 16, 22]. Despite prevailing recommendations against breastfeeding at that time, a small number of mothers, mostly born in African countries but living with HIV in North America, chose to breastfeed [7]. The most reported reasons for electing to breastfeed were perceived health benefits for the infant, social influences such as family expectations, the fear of disclosure of HIV status, and the desire for parent–infant bonding [7]. Over time, an increasing number of women living with HIV in high-income settings are choosing to breastfeed [22, 23].

Accommodating the interests of a growing number of parents with HIV in the US who are choosing to breastfeed and considering the low risk of HIV transmission with sustained viral suppression, the DHHS now strongly encourages shared decision-making between healthcare providers and parents regarding infant feeding in the context of perinatal HIV exposure [7]. Similarly, in May 2024, which was three months prior to the commencement of our survey, the American Academy of Pediatrics (AAP) revised its infant feeding guidelines to reflect evolving practices, placing particular emphasis on comprehensive counseling for parents with HIV who strongly desire to breastfeed [6]. This patient-centered counseling focuses on the potential risks of HIV transmission throughout the duration of breastfeeding. Furthermore, the AAP encourages support only in cases where specific criteria are met: ART initiated early in or prior to pregnancy, sustained viral suppression (defined as <50 copies per mL), demonstrated adherence to ART by the parent, antiretroviral prophylaxis (ARV) for the infant and continuous access to ART for the duration of breastfeeding [6].

Standardized counseling and effective shared decision-making regarding infant feeding require congruent perspectives among the multidisciplinary teams involved in caring for the parent and the infant. In this study, we observed a divergence in opinion among subspecialists. Fewer neonatologists than PID specialists in the US consider breastmilk from a parent living with HIV as a feeding option, particularly across certain geographical regions. In clinical practice, neonatologists play a more direct role than PID specialists in guiding infant feeding decisions. This discrepancy, along with differing viewpoints on infant feeding could present a significant barrier to the adoption of practice changes in certain regions. In a 2021 study conducted prior to the revision of feeding guidelines by DHHS, only 10% of surveyed US-based providers with experience in breastfeeding in HIV practiced at centers with an institutional protocol on the topic [24]. Similarly, our study found that only 35% of PID physicians and 28% of neonatologists reported practicing at centers with such protocols.

We surveyed the adoption of key components of the approach to the management of the breastfeeding dyad as proposed by the DHHS and affirmed by the AAP to minimize lactational HIV transmission. We found that among the minority of neonatologists who reported having a standardized discharge process, least commonly included components were the recommendation of breastfeeding duration (exclusive breastfeeding through the first 6 months), guidance on weaning from breastmilk, and instruction on breast care. Emergency provision of donor breast milk to prevent mixed feeding with formula was rarely practiced and may be limited by cost-related barriers. There is a critical need for more comprehensive in-hospital breastfeeding programs that provide holistic support to both the mother and the infant, thereby promoting optimal health outcomes for the breastfeeding dyad.

In the US, concerns regarding HIV transmission have long posed a significant barrier to recommending breastfeeding in the context of perinatal HIV exposure [16]. Despite recent shifts in clinical guidelines, our study reveals that this concern remains the strongest factor influencing clinical decision-making among PID specialists and neonatologists across all geographic regions. This barrier may be further contributing to the lack of standardized policies and procedures regarding breastfeeding in many centers. Additionally, we identified the absence of evidence-based data on the frequency of virologic testing in the breastfeeding infant as another significant barrier to breastfeeding support. Initial steps have been taken nationally to establish clinical guidance and promote standardization of care. In 2025, the DHHS Panel on Treatment of HIV during pregnancy and prevention of perinatal transmission updated recommendations providing more explicit guidance on the use of antiretroviral drugs. However, a consensus could not be reached on the extended use of ARV prophylaxis during the breastfeeding period, which is a critical aspect of clinical management. As a result, the panel emphasizes the need for open dialogue and a shared decision-making approach that carefully weighs the potential risks and benefits [16]. While providing guidance on infant ARV prophylaxis may contribute to the standardization of clinical management, it does not sufficiently address the persistent concerns regarding lactational HIV transmission among subspecialists. Enhanced data sharing, robust longitudinal follow-up within the US, and the establishment of a centralized registry to evaluate high-fidelity care models capable of ensuring near-zero transmission risk may help mitigate these concerns.

Several limitations should be considered when interpreting the findings of this study. We had a low response rate at only 10%. Although this falls within the standard response rates for subspecialists affiliated with both organizations and included a broad geographical distribution, it is possible that participants are not representative of all PID and neonatology subspecialists. Additionally, certain US states were underrepresented in the sample. To mitigate this limitation, survey data were stratified by geographic region during analysis. Nevertheless, this study represents the first comprehensive national assessment of clinical practice adoption including both PID specialists and neonatologists following the revision of US infant feeding guidelines in perinatal HIV exposure. It is also important to acknowledge that other key members of the multidisciplinary care team, such as adult infectious disease specialists, HIV physicians, immunologists, obstetricians, well baby nursery providers, general pediatricians and lactation consultants, play a critical role in the infant feeding decision-making process; however, they were not included in this survey. Importantly, some of these healthcare providers play an integral role in delivering prenatal counseling on infant feeding early in pregnancy. Further research is warranted to elucidate the unique barriers faced by these provider groups.

## CONCLUSIONS

Recommendations to support breastfeeding among parents with HIV have not been widely adopted in the US, with more PID subspecialists endorsing this practice than neonatologists. Perinatal HIV transmission risk remains a significant concern and an ongoing barrier. We believe that enhanced data sharing, robust longitudinal follow-up, and the establishment of a centralized registry within the US to evaluate high-fidelity care models capable of ensuring near-zero transmission risk may help mitigate these concerns. Next steps include an evaluation of implementation trends over the next year and an assessment of regional resource availability to support clinical adherence.

## Supplementary Material

ofaf607_Supplementary_Data

## Supplementary Data

Supplementary materials are available at *Open Forum Infectious Diseases* online. Consisting of data provided by the authors to benefit the reader, the posted materials are not copyedited and are the sole responsibility of the authors, so questions or comments should be addressed to the corresponding author.
