# Supplementary material for: Adoption of the Revised DHHS Guidelines on Breastmilk Feeding and HIV in the United States: Clinical Practices and Barriers
Source: Open Forum Infect Dis. 2025 Sep 27;12(10):ofaf607. doi: 10.1093/ofid/ofaf607 (PMC12527334; doi:10.1093/ofid/ofaf607)
Supplement: ofaf607_Supplementary_Data [file ofaf607_supplementary_data.pdf]

Breastfeeding in HIV exposure

We are conducting a national survey on breastfeeding in HIV exposure. In 2023, the Center for Disease Control and Prevention (CDC) revised breastfeeding policies to emphasize shared decision making and support for HIV positive individuals who desire to provide their own milk. The aim of this survey is to assess current physician practice patterns and identify barriers to implementation of this revised policy.

This survey contains 4 questions on demographics and a maximum of 8 questions on feeding practices and barriers. We anticipate the survey will take 5 minutes to complete.

This survey is for the purpose of research and tar ets attendin neonatolo , ediatric and adult infectious disease physicians. Our study has been approved by the [redacted]. No identifiable information will be collected. Multiple respondents are encouraged to participate from one institution. Answers will be collected via a secure REDCap, and individual answers will be kept strictly confidential. There is no direct benefit to participation, but we hope that this study will contribute to improving patient care in the future. There is no foreseen risk since all answers are kept confidential and are not identifiable. Participation is completely voluntary, and completion of the survey indicates willingness to consent.

Thank you.

Select your specialty

- ◯ Neonatology
- ◯ Adult Infectious Disease
- ◯ Pediatric Infectious Disease

How many years have you been practicing as an attending in your specialty?

- ◯ 0-5
- ◯ 6-10
- ◯ 11-15
- ◯ 16-20
- ◯ >20

In what state of the US do you practice?

____

What type of hospital system do you practice in?

- ◯ Academic/University-affiliated hospital system
- ◯ Non-academic/Non-university-affiliated system

In your center, is the pediatric infectious diseases team consulted when an infant is born to an HIV positive parent?

- ◯ Yes
- ◯ No

Do you provide inpatient consultation to infants of HIV positive parents after delivery?

- ◯ Yes
- ◯ No

Regarding the management of pregnant HIV positive patients, in which of the following stages of care are you involved? Please select all that apply

- ☐ Prenatal counselling
- ☐ Postnatal inpatient consultation
- ☐ Postnatal discharge and outpatient follow up
- ☐ None of the above

Do you discuss infant feeding options with HIV positive parents?

- ◯ Yes
- ◯ No

For infants born to HIV positive parents with undetectable viral load at delivery, what is your feeding recommendation? (Please select all that apply)

- ☐ Infant formula
- ☐ Breastmilk from the HIV positive parent (breastfeeding/expressed breast milk/chest feeding)
- ☐ Pasteurized human donor milk

Does your center have a protocol/ policy to guide infant feeding of breastmilk from an HIV positive parent?

- ◯ Yes
- ◯ No

Does your center have a standardized process for discharge and follow up of infants fed breastmilk from an HIV positive parent?	◯ Yes<br>◯ No
Does your center have a policy/protocol/guideline/standard practice for discharge and follow up of an HIV positive parent whose infant is being fed their own breastmilk?	◯ Yes<br>◯ No
For infants fed breastmilk from an HIV positive parent, which of the following is included in your hospital discharge plan? (Please select all that apply)	☐ Infectious disease specialist/HIV physician referral<br>☐ Timing of outpatient virologic testing of the infant<br>☐ Duration of breast/chest feeding<br>☐ Weaning off breastmilk<br>☐ Nursing technique and breast care<br>☐ Supplemental/emergency donor milk supplies to prevent mixed breastmilk and formula feeding<br>☐ Education on situations warranting discontinuation of breastfeeding<br>☐ Other
Please specify	__________________________________
For infants fed breastmilk from an HIV positive parent, which of the following is included in your hospital discharge plan or discussed during outpatient follow up visits? (Please select all that apply)	☐ Timing of outpatient virologic testing of the infant<br>☐ Frequency of outpatient virologic testing of the infant<br>☐ Duration of breast/chest feeding<br>☐ Weaning off breastmilk<br>☐ Avoidance of mixed breastmilk and formula feeding<br>☐ Education on situations warranting discontinuation of breastfeeding<br>☐ Others
Please specify	__________________________________
Which of the following is included in your discharge plan or discussed during outpatient follow up of HIV positive parents whose infants are fed their own breastmilk? (Please select all that apply)	☐ Timing of outpatient virologic testing of the HIV positive parent<br>☐ Frequency of outpatient virologic testing of the HIV positive parent<br>☐ Duration of breast/chest feeding<br>☐ Weaning off breastmilk<br>☐ Nursing technique and breast care<br>☐ Avoidance of mixed breastmilk and formula feeding<br>☐ Education on situations warranting discontinuation of breastfeeding<br>☐ Other
Please specify	__________________________________

Which of the following are barriers to your provision of support to HIV positive parents who intend to feed their infants their own milk? (Please select all that apply)

- ☐ Concern for risk of HIV transmission to the infant
- ☐ Lack of institutional policy/guideline
- ☐ Infectious disease specialists at my center do not support this practice
- ☐ Pediatricians/neonatologists at my center do not support this practice
- ☐ Lactation specialists at my center do not support this practice
- ☐ Limited access to Infectious disease/HIV physicians within my geographic area
- ☐ Lack of data on frequency of virologic testing of the breastfeeding HIV+ parent and infant
- ☐ Limited access to Donor breast milk as an option to prevent mixed breastmilk and formula feeding
- ☐ Other
- ☐ None

Please specify

____

Rank your top 3 barriers in order of importance (barrier 1 being the most important)

	Barrier 1	Barrier 2	Barrier 3
Concern for risk of HIV transmission to the infant	○	○	○
Lack of institutional policy/guideline	○	○	○
Infectious disease specialists at my center do not support this practice	○	○	○
Pediatricians/neonatologists at my center do not support this practice	○	○	○
Lactation specialists at my center do not support this practice	○	○	○
Limited access to Infectious disease/HIV physicians within my geographic area	○	○	○
Lack of data on frequency of virologic testing of the breastfeeding HIV+ parent and infant	○	○	○
Limited access to Donor breast milk as an option to prevent mixed breastmilk and formula feeding	○	○	○
Other	○	○	○

Please specify

____
